# Influence of Heat Treatment Temperature on Microstructure, Hardness and Sensitization of UNS S32205 Duplex Stainless Steel

**DOI:** 10.3390/ma17194715

**Published:** 2024-09-25

**Authors:** Pedro Victorio Caetano Abrantes Quadros, Jomar José Knaip Ribeiro, Bruna Corina Emanuely Schibicheski Kurelo, Oriana Palma Calabokis, Yamid E. Nuñez de la Rosa, Alba Regina Turin, Paulo César Borges

**Affiliations:** 1 Postgraduate Program in Mechanical and Materials Engineering, Universidade Tecnológica Federal do Paraná (UTFPR), Campus Ecoville, Curitiba 81280-34, PR, Brazil; jomarribeiro@alunos.utfpr.edu.br (J.J.K.R.); bruna_schibicheski@hotmail.com (B.C.E.S.K.); opalmac@libertadores.edu.co (O.P.C.); yenunezd@libertadores.edu.co (Y.E.N.d.l.R.); albaturin.2020@alunos.utfpr.edu.br (A.R.T.); 2 Postgraduate Program in Science/Physics, Universidade Estadual de Ponta Grossa, Campus Uvaranas, Ponta Grossa 84030-900, PR, Brazil; 3 Faculty of Engineering and Basic Sciences, Fundación Universitaria Los Libertadores, Bogotá 111221, Colombia

**Keywords:** UNS S32205, intergranular corrosion, DL-EPR, sigma phase (σ), thermal cycle

## Abstract

Improper thermal cycles on duplex stainless steels can lead to the formation of detrimental phases or alter the proportion of ferrite and austenite phases, thus influencing the material’s mechanical properties and corrosion resistance. Therefore, this study aimed to evaluate the effect of aging (at 850 and 950 °C) and solubilization (at 1000 and 1150 °C) thermal treatments on microstructure, indentation hardness, elasticity modulus, and susceptibility to intergranular corrosion of UNS S32205 duplex stainless steel. The sigma phase (σ) formation in the aged samples, with hardness values between 8 and 10 GPa, was confirmed. Furthermore, the pieces treated from 1000 °C upwards showed that increased temperature favored the formation of more equiaxial grains and the ferrite fraction growth. The thermal treatments barely affected the elasticity modulus of austenite and ferrite grains, increasing the hardness of ferrite. The effect of sulfuric acid concentration in the intergranular corrosion was evaluated. Also, the deconvolution of the corrosion curves permits the determination of the influence of the different phases in the corrosion performance. These tests revealed sensitization only at the σ phase grain boundaries in the samples treated at 850 °C in electrolytes containing H_2_SO_4_ 2.5 mol/L and HCl 1 mol/L. Although the treatment at 950 °C led to the σ phase formation, its higher corrosion resistance was ascribed to the lower volumetric fraction of this phase, its morphology, and its increased Cr mobility compared to the 850 °C treatment. Therefore, it was shown that the σ characteristics and the sulfuric acid concentrations are determining factors in the UNS S32205 intergranular corrosion resistance.

## 1. Introduction

Duplex stainless steels (DSSs) are metallic alloys that have a two-phase microstructure composed of approximately equal amounts of ferrite (α) and austenite (γ) phases. DSSs present higher mechanical and corrosion resistance when compared to other classes of stainless steels (SSs), such as ferritic and austenitic steels, providing high applicability due to the combination of these properties [1,2,3,4,5].

However, depending on the application of these alloys, when subjected to processes such as welding to manufacture parts and machinery or to thermal and thermochemical treatments, metallurgical changes associated with the proportions of the ferrite and austenite phases can be induced, as well as deleterious phase precipitation, such as sigma (σ), chi (χ), carbides (M_23_C_6_), or nitrides (CrN), which increase DSS susceptibility to corrosion [3,6,7,8]. Deleterious phases, such as σ, are rich in Cr and impoverish neighboring regions, thus making SS susceptible to intragranular corrosion [9,10,11,12].

In such a context, electrochemical techniques are employed to assess material corrosion resistance. Among such techniques, the double loop electrochemical potentiokinetic reactivation (DL-EPR) stands. Based on the ISO 12732 standard [13], this technique is characterized by a selective attack on the grain boundaries to identify Cr depletion in these regions based on the sensitization of the material. In addition, it allows the determination of the degree of sensitization (DOS) of stainless steels [13,14,15]. DOS enables the determination of the precipitation effect of Cr-rich deleterious phases on grain boundaries in the material intergranular corrosion [13].

The ISO 12732 standard [13] determines a solution composed of sulfuric acid (H_2_SO_4_) in a 0.5 to 2.5 mol/L concentration and the potassium thiocyanate (KSCN) depassivator, in a 0.001–0.05 mol/L range. However, studies in the literature report using different solutions to evaluate intergranular corrosion. Specifically, in DSS studies adopting the DL-EPR technique, researchers have applied different electrolyte solutions. Among those solutions, the adoption of sulfuric acid with thioacetamide (TA) [15,16] or with hydrochloric acid (HCl) [17,18], has stood out.

In such context, the authors Symniotis [19], Duret-Thual, Bonis and Crolet [17], and Gong et al. [20] evaluated different concentrations of the depassivator employed. However, few studies have investigated the effect of sulfuric acid concentration in DL-EPR tests on sensitivity to evidence of intergranular corrosion in microstructures obtained through different thermal cycles [17,21]. The parameters used in the reports found in the literature that explored DL-EPR tests on UNS S32205 (UNS S31803) are summarized below (Table 1). The table highlights the surface preparation, the polarization range, and the solutions used to evaluate the DSS susceptibility to intergranular corrosion. Therefore, the importance of examining the changes promoted by different thermal treatments in the microstructure of these materials is evident. Not only does the direct analysis of microstructure changes enhance understanding of DSS properties, but they also clarify their resistance to localized corrosion.

Therefore, there is a need to evaluate the changes promoted by different heat treatment temperatures in the microstructure of DSS and its resistance to localized corrosion. Furthermore, it can be highlighted that there is a lack of studies in the literature that explore different concentrations of sulfuric acid to determine the degree of sensitization of DSS with different percentages of σ, ferrite, and austenite subjected to different heat treatment temperatures, and the influence on dissolution peaks obtained by DL-EPR.

Taking all that into consideration, the objective of this study was to investigate the electrochemical behavior of UNS S32205 DSS subjected to distinct aging thermal treatments (850 and 950 °C) and solubilization (1000 and 1150 °C) employing the DL-EPR technique. Additionally, the DL-EPR curve deconvolution was carried out, which enabled a determination of the influence of the different phases in the material’s resistance to corrosion. Furthermore, X-ray diffraction (XRD), microscopy, and quantitative stereology of the phases revealed by the chemical attack were used in the microstructural characterization of surfaces and phases. Finally, the nanoindentation technique was used to analyze the nanomechanical behavior of the material surface.

## 2. Materials and Methods

### 2.1. Sample Preparation

The material used in the research was the UNS S32205 duplex stainless steel, supplied by the company Aperam South America (Belo Horizonte, Brazil). The chemical composition of the DSS obtained from the Aperam Research Center is presented below (Table 2).

Aging and solubilization thermal treatments were performed in the base material plates in 5 conditions, all followed by fast cooling in water. To better identify the conditions, a code was created for each sample (Table 3). The temperatures adopted in this study were selected based on previous studies on DSSs found in the literature [3,22,23,24,25].

Based on those studies, the temperatures were strategically selected to explore different aspects of the DSS microstructure. At 850 °C, greater precipitation kinetics of the σ phase was observed [3,22,23,25,26]. In comparison, at 950 °C a smaller σ fraction was evidenced due to its solubilization, followed by an increase in the volumetric percentage of the ferritic phase [22,27]. At 1000 °C, a two-phase microstructure was observed with a greater amount of γ in relation to α, while at 1150 °C, the predominance of the α phase on the γ phase was seen [24,28]. The choice of these different temperatures enabled a deeper understanding of the microstructural transformations that occurred in the DSS as a response to the thermal variations.

After the thermal treatment, 33 × 31 × 5 mm samples were cut using a water jet. Next, they underwent a rectification process and sanding with abrasive material varying from 220 to 600 mesh to be ready for the DL-EPR tests. For phase quantification and nanohardness mechanical characterization, additional sanding up to 1200 mesh was carried out, followed by polishing with alumina suspension (Al_2_O_3_) (1 µm) and diamond paste (1 µm). The preparation process was concluded with a chemical attack to reveal the material microstructure.

### 2.2. Microstructural Analysis

The XRD technique was used to identify the phases found in the microstructure in all conditions investigated. A diffractometer (XRD: Shimadzu, model XRD-7000, Shimadzu Corporation, Tokyo, Japan), with Copper incident radiation (CuK-α), was used, scanning between 30 and 55° (2θ) at 1°/min scanning rate, and angular step equal to 0.02°.

Immersion chemical attack was used to reveal the UNS S32205 DSS microstructure, using Behara reagent (80 mL H_2_O + 20 mL HCl + 1 g K_2_S_2_O_5_) for 30–40 s with samples BM, S1000, and S1150. Modified Behara (80 mL H_2_O + 20 mL HCl + 1 g K_2_S_2_O_5_ + 2 g ((NH_4_) HF_2_)) was used for 5 s with samples that presented the σ phase (S850, S850-2h, and S950) [22,25].

For the microstructural analysis, optical micrographs of the sample surfaces were obtained with enlargements from 50 to 2000×. The quantification of the UNS S32205 DSS primary (ferrite and austenite) and deleterious phases was carried out using the quantitative stereology technique aided by the ImageJ^®^ software (ImageJ v.1.53, Wayne Rasband, National Institute of Health, Bethesda, MD, USA). Forty top micrographs were analyzed after being obtained using optical microscopy (OM) (Olympus BX51RF, Olympus Corporation Shinjuku Monolith, Tokyo, Japan) with 1000× enlargement for each treatment condition, as performed by Magnabosco [23].

### 2.3. Nanomechanical Analysis

**Figure 1:**
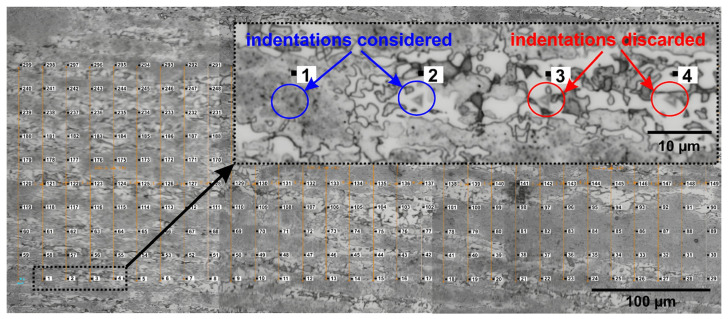
Matrix of 10 × 30 indentations with 20 µm spacing and 5 mN load performed on sample S850-2h. The details show that indentations (1 and 2) in the grain’s center were considered for calculating the average values of hardness and modulus of elasticity, and indentations (3 and 4) were discarded because they were close to grain boundary regions.

The nanoindentation technique was applied to determine the hardness and modulus of elasticity of grains of the austenite, ferrite, and σ phases. The measurements were carried out according to the ISO 14577-1 (2002) standard [26], and the Oliver and Pharr method [27], and the equipment applied in the tests was a nanoindenter (model ZHN/ZwickRoell, GmbH & Co. KG, Ulm, Germany). The samples previously chemically etched with Behara reagents to reveal the different phases present in the material were indented with a Berkovich indenter. In each sample, 299 indentations were arranged in 30 × 10 matrices with a 20 μm interval between indentations. The single-loading tests were performed in the central regions of the samples with a 5 mN load. Then, optical micrographs were obtained to separate the indentations related to each phase. Indentations close to the grain boundaries were discarded, and only indentations in the grain centers were considered to calculate the average hardness and modulus of elasticity. Figure 1 shows the indentation matrix of sample S850-2h.

As it is noted in Figure 1, where the indentation matrix of sample S850-2h is shown, indentations 1 and 2 were performed in the center of the austenite and σ phase grains, respectively, and were considered for the analysis of average hardness and elastic modulus. Indentations 3 and 4 were discarded and not considered in the average because they were in grain boundary regions.

### 2.4. Electrochemical Analysis

The DL-EPR technique was used to assess the electrochemical behavior of the material investigated. Intragranular corrosion tests were carried out using an EmStat^3+^ potentiostat (PalmSens BV, Utrecht, The Netherlands) assisted by the PSTrace 5.9 software (PalmSens BV, Utrecht, The Netherlands) in a three-electrode system containing the DSS sample as working electrode, one graphite auxiliary electrode, and a reference electrode, Ag/AgCl with KCl 3 mol/L. Three different electrolytes containing sulfuric and chloride acid were assessed, and in each test, the electrolyte volume used was ~70 mL at 25 ± 5 °C. The H_2_SO_4_ molarities selected were 0.5, 1.5, and 2.5 mol/L, while the HCl concentration was 1 mol/L in all tests. The parameters adopted in the electrochemical test were based on the ISO 12732 standard [13]. The open circuit potential (OCP) was initially measured for 300 s. The stabilization criterion was defined when the potential variation was below 1 mV/min. The scanning speed was 1.67 mV/s (6 V/h), as indicated in the standard.

After that, polarization started in an anodic direction from a potential equal to −0.1 V versus the corrosion potential (E_corr_) recorded from the OCP up to the 0.3 V potential. Next, the text scanning direction was reversed to a cathodic direction until returning to the initial potential. All the potentials quoted in this work refer to Normal Hydrogen Electrode (NHE). The maximum activation (I_p_) and reactivation (I_r_) current densities were obtained and used for the DOS calculation using the (I_r_/I_p_) ratio.

The deconvolution was performed with Gaussian fitting to analyze each phase curve separately during the activation and reactivation. The number of fitting peaks was selected so that the weighted residue between the original signal and the sum of the fitted peaks became less than 0.01.

Finally, for the surface evaluation after the DL-EPR tests, the scanning electron microscopy (SEM) (Zeiss, model EVO MA 15, Carl Zeiss Ltd., Cambridge, UK), and energy dispersive X-ray spectroscopy (EDS) (Oxford, X-Max of 20 mm^2^, Wiesbaden, Germany) techniques were used.

## 3. Results and Discussion

### 3.1. Microstructural Characterization

**Figure 2:**
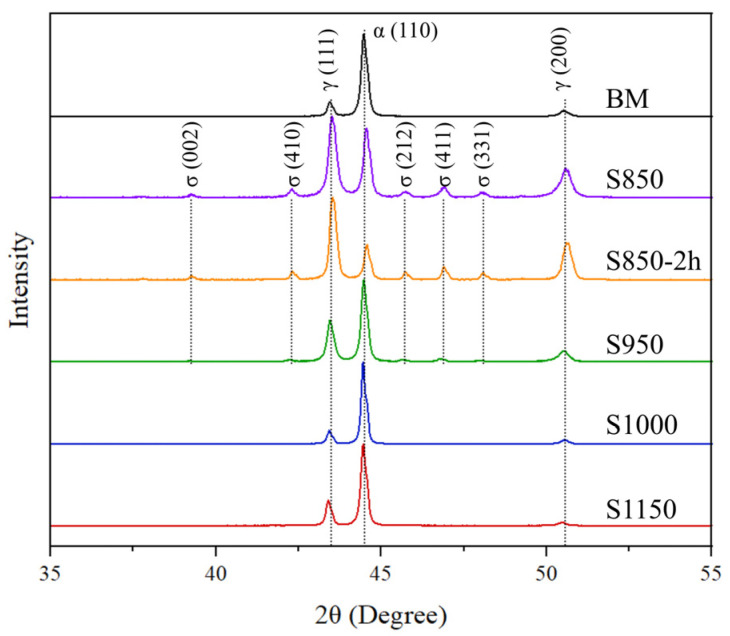
Diffractograms of the UNS S32205 DSS base metal after thermal treatments from 850 to 1150 °C for 30 min or 2 h.

The diffractograms of the samples with and without thermal treatment in the 35 to 55° (2θ) band are shown below (Figure 2). This is the point where the σ peaks were observed. The analysis of the diffractograms enabled the identification of the presence of peaks referring to the austenite phases (γ) for 2θ of approximately 43.4, 50.5, 73.4, 90.2, and 95.5°, and approximately 44.5, 82.1, and 98.4° for the ferrite (α) phase, in all conditions investigated. It was also observed that the secondary phase σ peaks only occurred in the S850, S850-2h, and S950 conditions, in the positions 39.4, 42.4, 45.7, 46.9, and 48° approximately. No peak of the χ or γ_2_ phases_,_ or any other phase, was identified. Such values were confirmed by the results found in other studies reported in the literature [18,26]. It seems relevant to emphasize that the difficulty related to the identification of other deleterious phases must be due to their low volumetric fraction [29,30]. However, this does not necessarily mean they were not present in the material [22,23].

**Figure 3:**
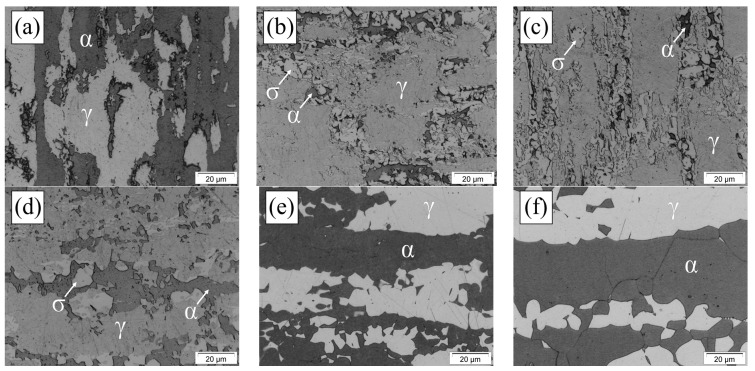
Micrographs of the UNS S32205 DSS normal section, obtained using OM in the conditions: (**a**) BM, (**b**) S850, (**c**) S850-2h, (**d**) S950, (**e**) S1000, and (**f**) S1150 (Attack: Behara for 30 s in the conditions (**a**,**e**,**f**); and modified Behara for 5 s in the conditions (**b**–**d**).

The UNS S32205 DSS images obtained using optical microscopy are presented below (Figure 3). The micrographs show the presence of a γ phase identified by the lighter color in the images (Figure 3a,e,f), obtained in the BM, S1000, and S1150 conditions, and by the intermediate color in the S850, S850-2h, and S950 samples (Figure 3b–d). When the σ phase is present, it is identified by the lighter color. The σ precipitation at 850 and 950 °C is notably found in other reports of aging thermal treatments in duplex and super duplex steel [3,22,23,25,31,32].

**Figure 4:**
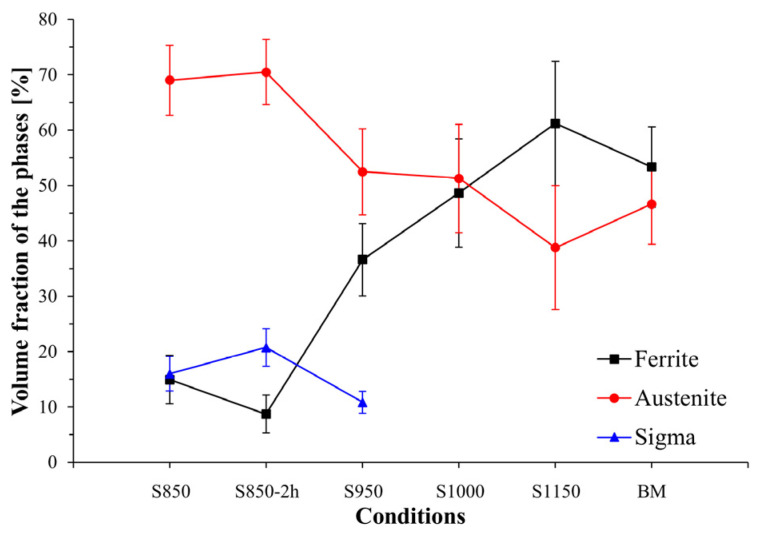
Volumetric percentage of α, γ, and σ phases found in the UNS S32205 DSS base metal microstructure and after thermal treatments from 850 to 1150 °C, for 30 min or 2 h.

Dos Santos and Magnabosco [22] reported not having observed the presence of the σ in thermal cycle temperatures over 1000 °C in UNS S31803 DSS. This absence is consistent with the phase diagrams, which predict that the σ is unstable over 985 °C and might dissolve at that temperature [29]. In addition, as expected, we observed that at 1150 °C, ferritic and austenitic grains became larger and more equiaxial when compared to temperatures below 1000 °C, or the BM condition. The results obtained for phase quantification in the normal section in the conditions investigated are presented below (Figure 4).

**Figure 5:**
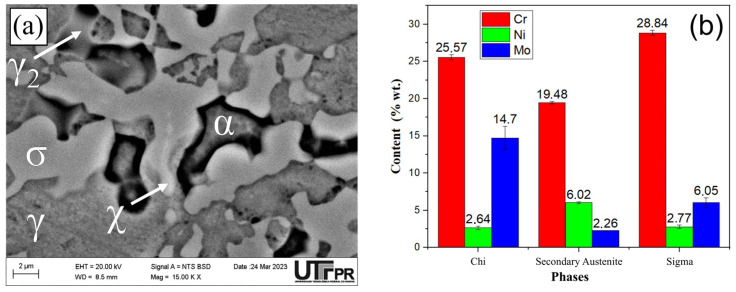
(**a**) Microstructure, obtained by SEM, of DSS UNS S32205 treated by aging at 850 °C for 30 min, and (**b**) Cr, nickel, and molybdenum content (wt.%) of χ, secondary austenite, and σ phases obtained by EDS microanalysis.

The ferrite, austenite, and σ percentage variation in the samples investigated (Figure 5) revealed that the highest percentage of the σ phase was found in the conditions subjected to 850 °C, thus indicating greater σ precipitation kinetics at that temperature. The σ percentage reduction with increased temperature is associated with this phase’s lower chemical stability [22]. In the S850, S850-2h, and S950 conditions, the austenite volumetric fraction prevailed in the microstructure of the material investigated. Such behavior might be associated with the precipitation of intermetallic compounds, such as the formation of σ or γ_2_ phases from the ferrite eutectoid decomposition, thus reducing the α phase percentage in those conditions [22,23].

Studies have identified the treatment time’s direct influence on the σ precipitation in DSSs [22,30,32]. Gao et al. (2018) [32] investigated the microstructure and corrosion resistance of the hot and cold rolled 2205 steel, aged using different treatment times at 850 °C. Increased σ fraction was observed as a function of time. After 60 min, σ volumetric fractions were observed in the (12.9–14.9) ± 0.2% band. Considering 2 h of thermal treatment, the σ volume increased to approximately (19.5–22.1) ± 0.2%. Moreover, those authors also observed the formation of χ and γ_2_ phases. 

Dos Santos and Magnabosco (2013) [33] studied the formation of deleterious phases from aging treatments at 850 °C at different times. They reported a decrease in the ferrite volume fraction from 40.6 ± 2.2% to 17.5 ± 2.9% and a significant increase in the σ content of up to 22.1 ± 4.02% in samples aged between 5 min and 1 h [24]. This volumetric variation of ferrite and σ indicates that the σ formation occurred via nucleation and growth from ferrite since the austenite fraction (the amount of austenite considered is the sum of the primary austenite found in the base metal and the secondary austenite (γ_2_) formed during the aging thermal treatment)^0^ remained almost constant (from 59 ± 2.2% to 60.4 ± 4.95%).

The conditions that underwent thermal treatment at over 1000 °C showed a significant volumetric percentage of the α phase, with the lowest γ volumetric fraction found in the condition treated at 1150 °C. This behavior can be ascribed to the allotropic transformation of the γ phase into α at higher temperatures, which agrees with the findings reported by Zhang et al. [24].

The microstructure obtained for the S850 condition showed the presence of ferrite (α), austenite (γ), σ, χ, and secondary austenite (γ_2_) phases (Figure 5a). The chemical composition results of the main elements (Cr, Ni, and Mo) found in the χ, secondary austenite, and σ phases are shown below (Figure 5b).

The chemical composition of the χ phase presented less Cr and more Mo than the σ phase. The Mo presence, a heavier element, conferred the χ phase a brighter appearance in the image obtained using backscattered electrons [22,26,29,34,35]. Regarding the S850-2h and S950 conditions, χ or γ_2_ phases were not identified with the EDS technique, which might indicate that the consumption of those phases with the aging treatment increased temperature and time, as reported in other studies [22,28]. Dos Santos and Magnabosco [22] employed thermodynamic simulation and found that the χ is not an equilibrium phase, which might lead to its consumption with increased aging time.

### 3.2. Hardness and Elastic Modulus Measurements

**Figure 6:**
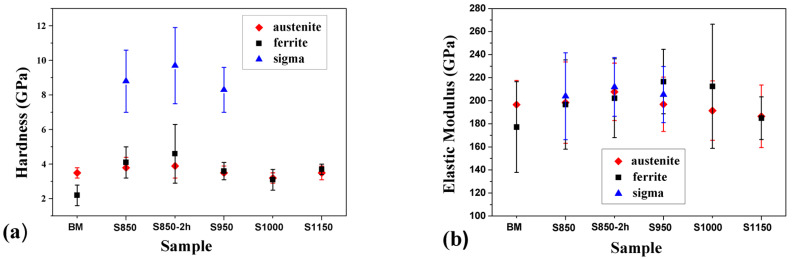
(**a**) Hardness and (**b**) Elastic Modulus of the BM and treated samples of duplex stainless steel obtained by nanoindentation.

When investigating the hardness and Elastic Modulus of the BM and treated samples of DSS obtained by nanoindentation with single loading and 5 mN load, the BM sample presented a hardness of 2.2 ± 0.6 GPa for the ferrite grains and 3.5 ± 0.3 GPa for the austenite grains (Figure 6).

In the samples S850, S850-2h, and S950, where the σ is formed, the hardness of the grains of this phase varied between 8.3 GPa (sample S950) and 9.7 GPa (sample S850-2h). The higher hardness value of the σ obtained in the S850-2h condition may be related to the increase in treatment time, which has increased the proportion of the σ (as observed in Figure 4) and caused an increase in grain size compared to the other treatment conditions, as observed in others research studies on duplex steels [8,34,35].

Due to the high hardness of the grains of the σ, the presence of this phase in duplex steel 2205 can increase wear resistance. However, depending on the sliding rates and the distribution of the grains of the σ, an increase in wear rates also can be observed because the σ has a brittle behavior [18].

After the thermal treatment at 850 °C for 30 min, the σ formed in the S850 sample with an 8.8 ± 1.8 GPa hardness. These hardness values agree with values for these phases obtained in a previous study [25]. In other works, where in addition to the formation of the σ, there was also the formation of the χ and precipitates of Cr nitrides and carbides, the hardness of the grains of the austenite and ferrite phases became higher, between 6 GPa and 7 GPa [35,36].

In the S850 sample, there was an increase in the hardness of the ferrite phase after the thermal treatment when compared to the BM sample. Considering the dispersion in the hardness values, the hardness of the grains of α and γ phases remained close in the S850, S850-2h, and S950 samples, which is the temperature range where the σ precipitated. In the S1000 condition, which has a volumetric fraction between the ferrite and austenite phases close to each other, there was no more σ formation, and the hardness values of the ferrite and austenite phase grains were very close. In a treatment temperature condition of 1150 °C, the average grain hardness values became lower for the austenite phase at the ferrite phase.

The variation in hardness in the grains of different phases is related to different microstructures, the presence of precipitates, variations in grain size, and the distribution of residual stresses caused by quenching [37]. The hardness results were close to those observed in another study using the same material but on a different cutting plane [25]. It is also worth highlighting that the grains of the ferrite and σ phases are much smaller than the austenite grains. This may influence the plastic deformation field depending on the proximity of the indentations to the grain boundaries of different phases.

Figure 6b shows a significant dispersion in elastic modulus values. Considering this dispersion, the elastic modulus values are statistically similar regardless of the treatment. This similarity is because the elastic deformation field is broader than the plastic deformation field, allowing neighboring grains to influence the elastic modulus measurements even with small loads. Therefore, the modulus values represent an average behavior between the grains of the different phases, contributing to the measurements’ dispersion. Considering the dispersion in the values, the elastic modulus was close to values found in the literature for duplex stainless steel [25,37,38,39,40,41].

### 3.3. Electrochemical Characterization

**Figure 7:**
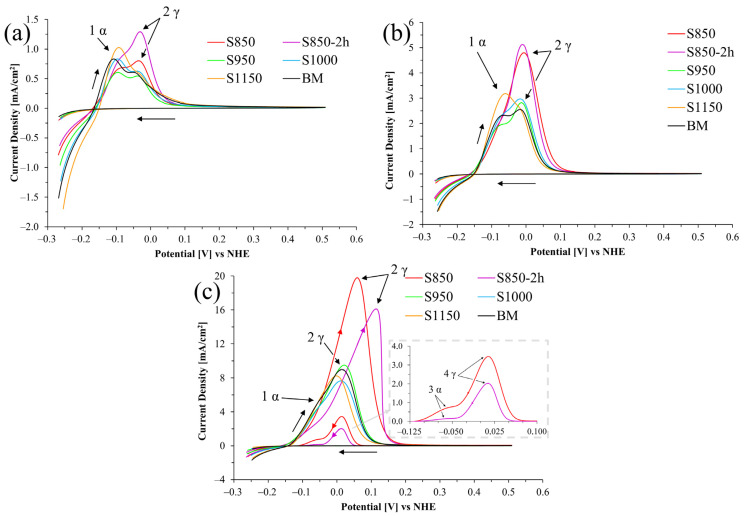
Graphs of the DL-EPR tests carried out in the UNS S32205 DSS in a hydrochloric acid medium (HCl 1 mol/L) with sulfuric acid (H_2_SO_4_) in the following concentrations: (**a**) 0.5 mol/L, (**b**) 1.5 mol/L, and (**c**) 2.5 mol/L.

The results of susceptibility to intergranular corrosion (Figure 7) are shown using the DL-EPR test polarization curve graphs for the thermal treatment conditions in different sulfuric acid solutions (0.5, 1.5, and 2.5 mol/L).

The current density increased when the sulfuric acid concentration in the electrolyte increased. This suggests that the medium becomes more active and corrosive with the increased H_2_SO_4_ concentration in the electrolyte used in the electrochemical test. Duret-Thual, Bonis, and Crolet [17] reported that the higher sulfuric acid molarity increases the activation and reactivation currents for the DL-EPR test.

Only the S850 and S850-2h conditions (Figure 7c) showed a current density peak during reactivation, resulting in similar graphs to those determining the degree of sensitization (DOS), according to the ISO 12732 standard [13]. The increased reactivation current peak is associated with the greater volumetric fraction and the deleterious σ phase morphology difference, which is responsible for the Cr depletion at the phase boundaries, with consequent reduction in the intergranular corrosion resistance [42,43]. It seems relevant to highlight that in the S850 condition, smaller grain size and larger boundary area were observed for the σ compared to the S950 condition (Figure 3b–d). Such characteristics increased the grain boundary area and the Cr depletion region, thus creating a larger extension with electrochemical difference potential. This phenomenon tends to favor the material’s susceptibility to intergranular corrosion. Such behavior was confirmed by Lo, Kwok, and Chan [11], who demonstrated that the morphology and evolution of the σ have a significant influence on the results obtained using the DL-EPR test. Those authors pointed out that the σ formation promotes Cr depletion in the primary austenite close to the σ particles, thus compromising the efficacy of the oxide layer as a protection barrier during the reactivation scanning. In addition, the study by Lo, Kwok, and Chan [11] emphasized that the σ/austenite interfaces became more predominant (with smaller grain sizes and a larger volumetric fraction of the σ) in aging treatments carried out at lower temperatures, which resulted in high DOS values.

When analyzing the DL-EPR curves (Figure 7) of the BM, S950, S1000, and S1150 conditions, it is observed that the current variation in these four conditions remained within the standard deviation intervals recorded during the tests, which suggests similarity in the electrochemical behavior of such samples. Notably, the σ precipitation observed in the thermal treatment at 950 °C did not lead to a significant loss in the 2205 duplex stainless steel resistance. This phenomenon might be ascribed to the lower volumetric fraction of the σ observed, its more regular morphology, and the Cr increased mobility with the aging increased temperature. This explains the reduced depletion of this element with consequent improvement in the intergranular corrosion resistance. This finding reinforces the understanding that the 2205 duplex stainless steel resistance to corrosion can be preserved upon certain thermal treatment conditions, even in the presence of potentially unfavorable precipitated phases. Comparatively, when evaluating the aged 2707 hyper duplex stainless steel, investigated by Sun et al. [41], the DL-EPR polarization curves (in a H_2_SO_4_ 2 mol/L + HCl 1 mol/L medium) were similar to those of the base material and that after aging at 900 °C for 30 min, even presenting the deleterious σ and Cr nitride deleterious phases. Based on the report put forward by Rezende et al. [42] on the solubilized UNS S31803 duplex stainless steel, the base metal results, and in the condition solubilized at 1100 °C for 30 min, they also showed similar curves after the DL-EPR tests (in a H_2_SO_4_ 2 mol/L + HCl 1 mol/L medium).

Another relevant observation obtained from the analysis of the polarization curves (Figure 7) was the identification of two distinct current activation peaks, marked by arrows 1 and 2 in all tests performed. Two current peaks were also found in the reactivation curves of the S850 and S850-2h samples, indicated by arrows 3 and 4, in an H_2_SO_4_ 2.5 mol/L + HCl 1 mol/L medium.

According to Lee, Jeon, and Park [43], the current peak located in a higher potential (arrow 2) is associated with the austenite dissolution. In contrast, the lower potential peak (arrow 1) is associated with ferrite dissolution. Furthermore, Lo et al. [21] reported that for an HCl concentration of 1 mol/L, the austenite dissolution current peak usually tends to be more evident and dislocate to a nobler potential when the electrolyte becomes more aggressive (Figure 7c). Moreover, this might justify the behavior of all curves in Figure 7, which showed current peaks located in increasingly higher potentials.

This study also evidenced the direct influence of the H_2_SO_4_ concentration (0.5 and 1.5 mol/L) in the current peaks 1 and 2 intensity. The increased H_2_SO_4_ concentration increased the intensity of the peaks referring to the austenite phase (arrow 2) in all conditions.

**Figure 8:**
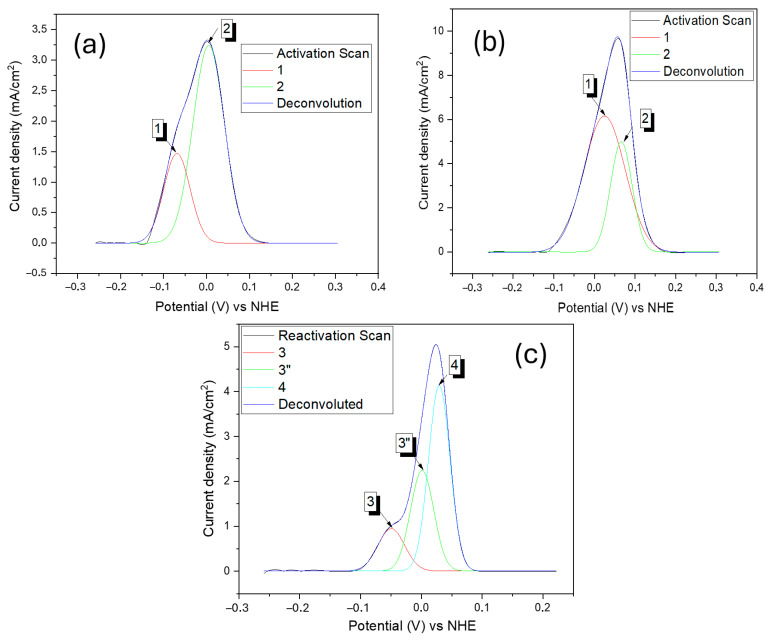
Deconvolutions of dissolution peaks obtained from the DL-EPR curves carried out in H_2_SO_4_ 2.5 mol/L and HCl 1 mol/L: (**a**) S1000—activation scan, (**b**) S850—activation scan, and (**c**) S850—reactivation scan.

The ferrite and austenite dissolution potentials are critical factors in the study of metallurgic processes. These potentials were obtained in all conditions during the activation and reactivation curves (Figure 7). Seeking better understanding and accuracy, we used the deconvolution technique to separate mixed signals (dissolution potential of each phase) of scanning during the tests in their components. The ferrite and austenite dissolution potentials obtained by this process (Table 4) are presented below, and how the deconvolution process was made is shown in Figure 8.

The dispersion of the potentials presented by the α and γ phases was smaller in the tests carried out at concentrations of 0.5 and 1.5 mol/L of H_2_SO_4_ in all conditions. A higher standard deviation was observed for the H_2_SO_4_ 2.5 mol/L + HCl 1 mol/L electrolyte, especially for the peak referring to austenite. Similar potentials versus NHE were found in other studies using DSS and super duplex stainless steel (SDSS) tested in media composed of hydrochloric and sulfuric acid, with dissolution potentials between −4 and 21 mV for austenite and between −76 and −50 mV for ferrite [21,43,44].

Furthermore, it seems relevant to emphasize that the deconvolutions revealed the existence of three peaks in the reactivation period in the S850 and S850-2h conditions. The additional peak may be associated with the material matrix corrosion and intergranular corrosion intensification. Wu and Tsai [45], when studying a UNS N06600 alloy (Inconel 600 alloy) employing the single loop electrochemical potentiokinetic reactivation technique (SL-EPR), observed a polarization curve associated with three individual peaks. According to those authors, such peaks could be associated with matrix, intergranular, and pitting corrosion. In this study, localized pitting corrosion was not observed. However, at the matrix composed of ferrite and austenite in the conditions in which there was the presence of a reactivation peak, we observed that the boundaries of the σ suffered intense corrosion. The austenitic phase also showed increasingly severe corrosion with the sulfuric acid concentration increase in the electrolyte, which will be discussed later.

These findings suggest that the increase in the H_2_SO_4_ molarity in the electrolyte increased the intensity of the intergranular corrosion of both the σ phase and the γ phase, and only at a 2.5 mol/L sulfuric acid concentration was the electrolyte aggressive enough to cause intergranular corrosion at the boundaries of the austenite grains, presenting a reactivation peak in the DL-EPR graphs in the S850 and S850-2h conditions.

**Figure 9:**
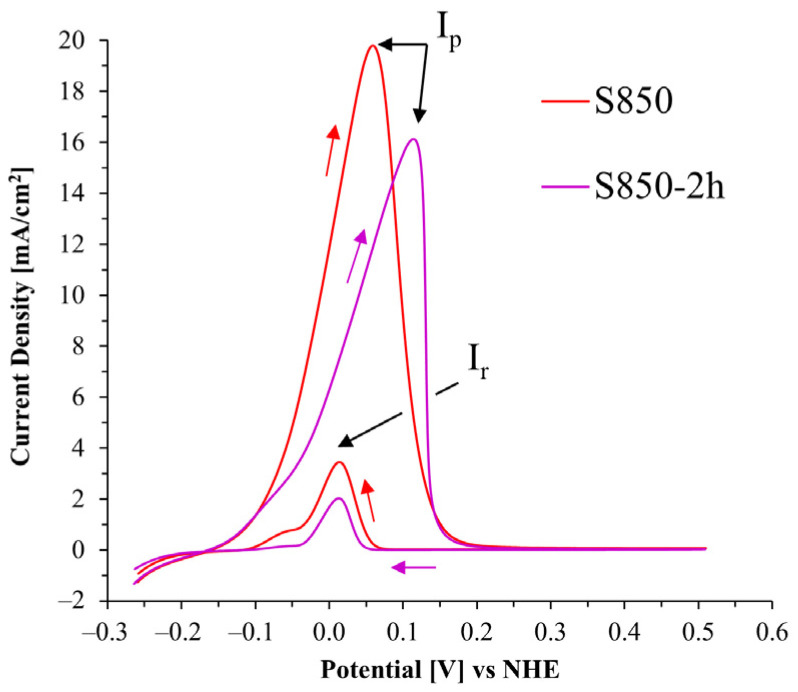
Current density graphs according to the potential in the S850 and S850-2h conditions after the DL-EPR test in an electrolyte containing H_2_SO_4_ 2.5 mol/L and HCl 1 mol/L.

Furthermore, it seems necessary to highlight that most studies in the literature investigating the DL-EPR tests developed in DSS analyzed two-phase materials. However, this study presents some conditions with a third phase in significant amounts (σ). The degree of sensitization was determined from the DL-EPR curves obtained from the conditions that exhibited activation and reactivation peaks. These were those treated at 850 °C for 0.5 h and 2 h (Figure 9) in the 2.5 mol/L sulfuric acid solution.

The sensitization parameters obtained from the DL-EPR curves in the S850 and S850-2h conditions are presented below (Table 5). According to the ISO 12732 standard [13], DOS values above 0.05 categorize the material as sensitized. Despite the significant difference in the DOS mean value between the two conditions, the ratio value, which is a measure of the degree of sensitization related to the study condition, is within the same interval in both conditions. This suggests that the degree of sensitization is similar for both conditions despite the difference in the DOS mean value.

**Figure 10:**
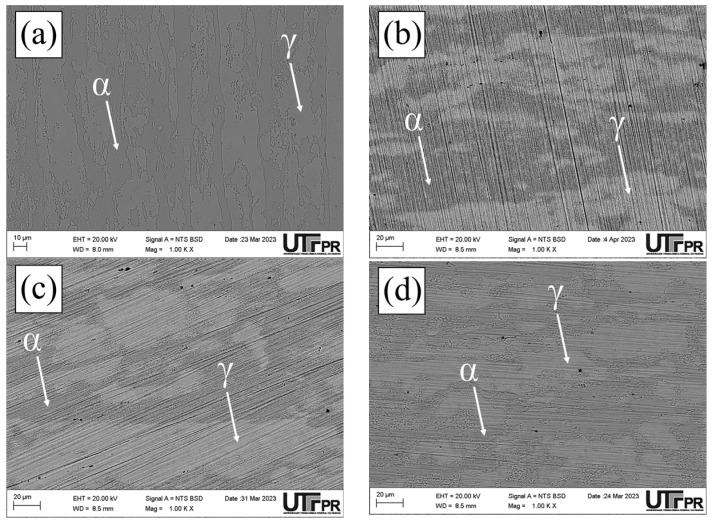
SEM micrographs of the UNS S32205 DSS in the BM condition after a chemical attack with Behara reagent for 30 s (**a**) and after the DL-EPR test in an electrolyte containing HCl 1 mol/L and H_2_SO_4_ at the following concentrations: (**b**) 0.5 mol/L, (**c**) 1.5 mol/L, and (**d**) 2.5 mol/L.

**Figure 11:**
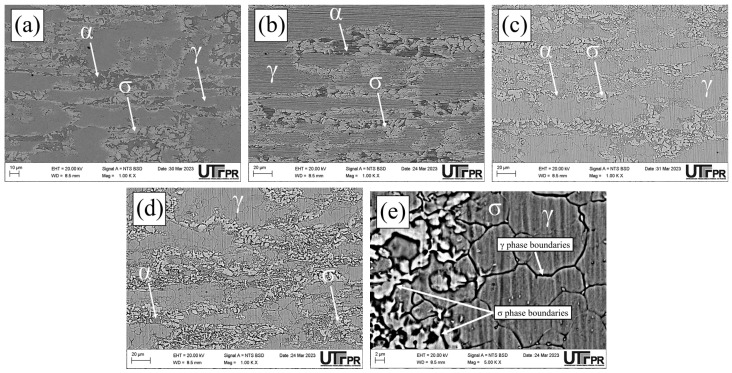
SEM micrographs of the UNS S32205 DSS subjected to thermal treatment at 850 °C for 30 min after chemical attack with modified Behara reagent for 5 s (**a**) and after the DL-EPR test in electrolyte containing HCl 1 mol/L and H_2_SO_4_ at the following concentrations: (**b**) 0.5 mol/L, (**c**) 1.5 mol/L (**d**,**e**) 2.5 mol/L.

**Figure 12:**
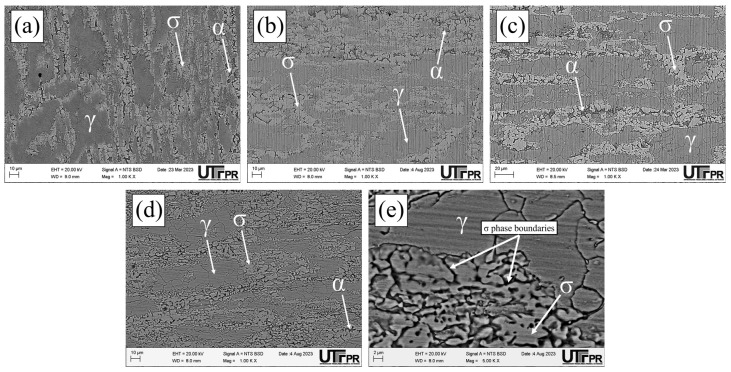
SEM micrographs of the UNS S32205 DSS subjected to thermal treatment at 850 °C for 2 h after a chemical attack with modified Behara agent for 5 s (**a**) and after the DL-EPR test in an electrolyte containing HCl 1 mol/L and H_2_SO_4_ at the following concentrations: (**b**) 0.5 mol/L, (**c**) 1.5 mol/L, (**d**,**e**) 2.5 mol/L.

**Figure 13:**
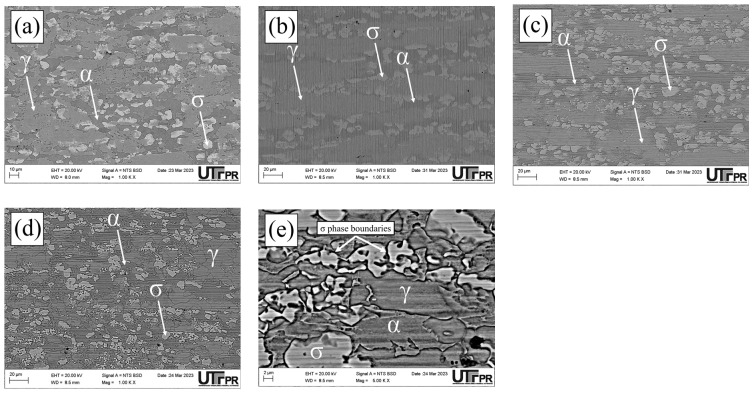
SEM micrographs of the UNS S32205 DSS subjected to thermal treatment at 950 °C for 30 min after chemical attack with modified Behara reagent for 5 s (**a**) and after the DL-EPR test in electrolyte containing HCl 1 mol/L and H_2_SO_4_ at the following concentrations: (**b**) 0.5 mol/L, (**c**) 1.5 mol/L, (**d**) 2.5 mol/L, and (**e**) 2.5 mol/L.

**Figure 14:**
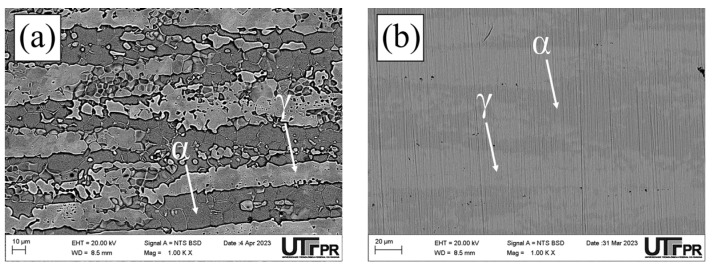

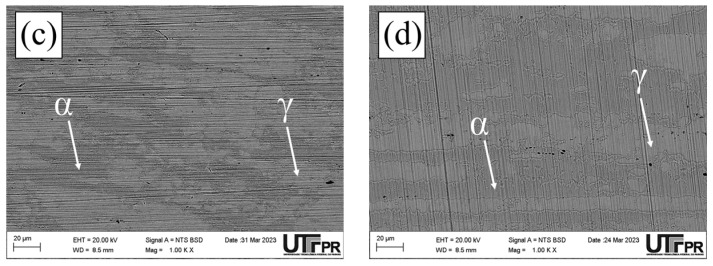
SEM micrographs of the UNS S32205 DSS subjected to thermal treatment at 1000 °C for 30 min after a chemical attack with Behara reagent for 40 s (**a**), and after the DL-EPR test in an electrolyte containing HCl 1 mol/L and H_2_SO_4_ at the following concentrations: (**b**) 0.5 mol/L, (**c**) 1.5 mol/L, and (**d**) 2.5 mol/L.

**Figure 15:**
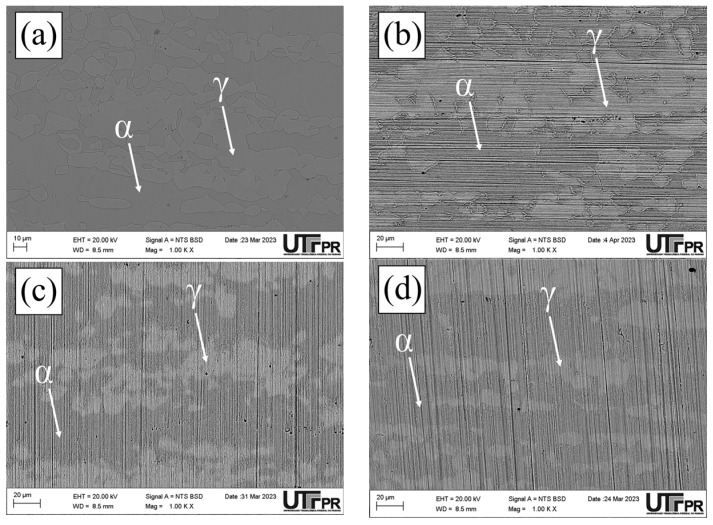
SEM micrographs of the UNS S32205 DSS subjected to thermal treatment at 1150 °C for 30 min after the chemical attack with Behara reagent for 30 s (**a**), and after the DL-EPR test in an electrolyte containing HCl 1 mol/L and H_2_SO_4_ at the following concentrations: (**b**) 0.5 mol/L, (**c**) 1.5 mol/L, and (**d**) 2.5 mol/L.

Micrographs of the normal section of the UNS S32205 duplex stainless steel before (only chemically attacked) and after the DL-EPR tests are presented in all conditions studied in different H_2_SO_4_ solutions (0.5, 1.5, and 2.5 mol/L) with the addition of 1 mol/L HCl (Figure 10, Figure 11, Figure 12, Figure 13, Figure 14 and Figure 15). Firstly, pit nucleation was not observed after the DL-EPR tests in any of the studied conditions, which allowed the use of the present results to analyze the susceptibility to intergranular corrosion of the material. The BM condition (Figure 10) showed that the sandpaper scratches resulting from the metallographic preparation persisted after the DL-EPR test in the electrolyte’s three concentrations of sulfuric acid. We also observed that the scratch became less prominent as the electrolyte became more aggressive, which is related to greater corrosion with the increased H_2_SO_4_ concentration in the electrochemical medium. Furthermore, no indication of attack promoted at the grain boundaries was observed after the DL-EPR test, which explains the lack of a reactivation current peak for the polarization curves. Rezende et al. [42], when investigating the SAF 2205 DSS, did not identify intergranular corrosion for that material in similar conditions to the ones of this study in a H_2_SO_4_ 2 mol/L + HCl 1 mol/L electrolyte.

The micrographs of the S850 condition revealed the presence of the σ (Figure 11). A more severe attack on the σ boundaries and a revelation of the austenitic boundaries of the austenite “islands” were observed with the increased H_2_SO_4_ concentration in the electrochemical medium (Figure 11c–e). These results can be correlated with the current peaks observed in the reactivation curves of the DL-EPR test (Figure 9). A similar behavior was observed in the S850-2h condition (Figure 12).

Analyzing the main surface changes after the DL-EPR tests on S850 and S850-2h samples in the 1.5 and 2.5 mol/L H_2_SO_4_ solutions, we observed that the boundaries of the σ phase became more explicit, indicating more intense corrosion provoked by these sulfuric acid concentrations when compared to the H_2_SO_4_ 0.5 mol/L condition. Alvarez et al. [46] investigated the UNS S32750 SDSS thermally treated at 850 °C for times varying from 5 to 90 min and used a H_2_SO_4_ 2.5 mol/L + KSCN 0.02 mol/L + NaCl 1 mol/L electrolyte for the DL-EPR tests. Del Abra-Arzola et al. [18] investigated the AISI 2205 DSS, treated for 30 min at 850 °C, and performed DL-EPR tests in H_2_SO_4_ 2 mol/L and HCl 1 mol/L. Both studies reported that, after the corrosion tests, the deleterious phase boundaries, mainly the σ, suffered more intense corrosion when compared to the other phases found.

In the S950 condition (Figure 13d), grain boundaries in both the σ phase and the γ phase were attacked mainly during the corrosion tests. However, in the austenite “islands” the austenitic grain boundaries appeared less corroded when compared to those in Figure 11c,d and Figure 12c,d. This can be confirmed by the polarization curves of the DL-EPR tests, where the S950 condition presented a lower current than those shown by the S850 and S850-2h conditions. Therefore, this difference in the corrosion intensity in activation curves reveals the seriousness of the corrosion attack in the γ phase boundaries. Similar intergranular corrosion images were verified in the study reported by Zanotto et al. [47] when investigating LDX 2101^®^ DSS treated at 650, 750, and 850 °C. Those authors observed that there was also the presence of an attack in the austenite grain boundaries despite the DL-EPR test having not presented a current peak in the reactivation curve.

Furthermore, it highlights that in addition to the σ volumetric fraction, the thermal treatment temperature is also a determining factor in the greater susceptibility to intergranular corrosion. As previously observed in the DL-EPR curves, the greater resistance to intergranular corrosion in the S950 condition might be related to the lower volumetric fraction of the σ and the smaller grain boundary area due to its more regular morphology, thus reducing the boundary areas between the σ and the matrix, that is, the smaller grain boundary area poor in Cr. The aging temperature also helps the recovery of intergranular corrosion resistance for increasing Cr diffusion, thus favoring its mobility in the matrix to regions depleted of this element.

Finally, we observed that the S1000 and S1150 samples showed electrochemical behavior similar to that of the BM sample (Figure 14 and Figure 15), where no sensitized grain boundaries were detected. Such results are related to the presence of a two-phase structure (α and γ), and electrochemical behavior similar to that of the polarization curves. The discrepancy of volumetric values in the ferrite and austenite phases in the BM, S1000, and S1150 samples was not identified as a determining factor for susceptibility to intragranular corrosion.

**Figure 16:**
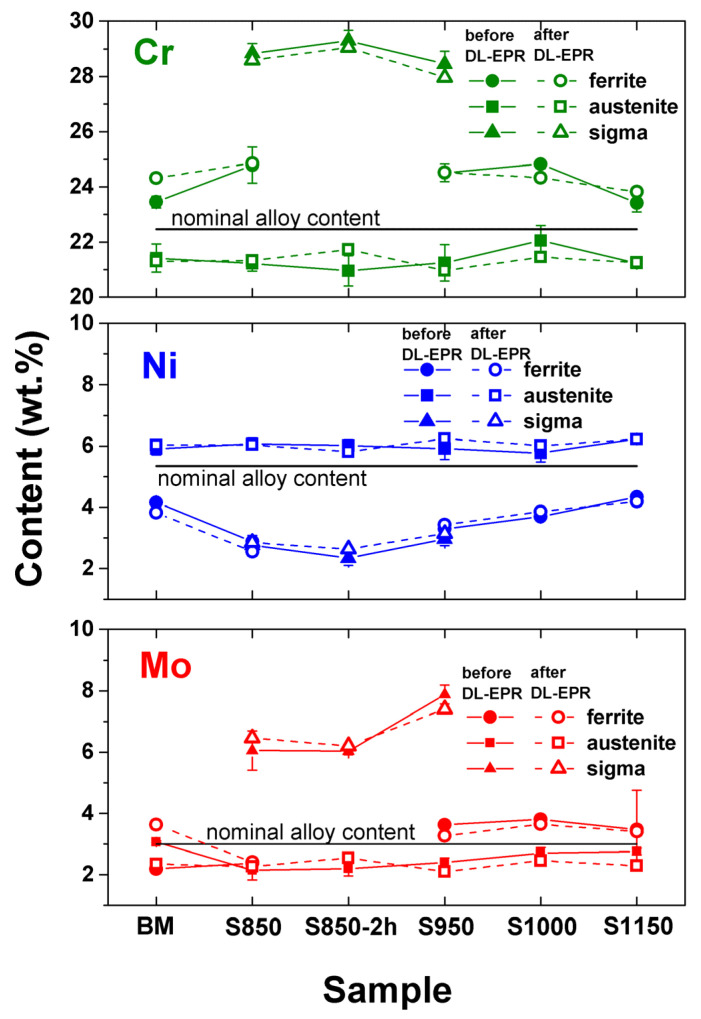
Cr, nickel, and molybdenum content (wt.%) of each phase in the studied conditions of DSS UNS S32205 samples thermally treated from 850 to 1150 °C before and after DL-EPR tests.

The alloy nominal composition and the results of the semiquantitative chemical composition of the phases before and after the corrosion test, determined using the EDS technique, are presented below (Figure 16).

The results obtained revealed that the ferrite phase, when compared to the austenite phase and, except for the BM condition, showed a higher percentage in weight of the alphagenic elements Cr and molybdenum (Figure 16). Meanwhile, the austenite presented a chemical composition with a higher percentage in weight of the gamagenic element nickel. Reports by Gong et al. [20], Zhang et al. [24], and Silva et al. [48] presented similar results for these phases. Moreover, both the α phase and the γ phase presented chemical compositions similar to those found in the literature [9,48,49].

In addition, the σ phase showed a chemical composition of around 28% Cr, 3% Ni, and 7% Mo. Likewise, Sieurin and Sandström [3], and Cavalcanti [27], when studying UNS S32205 and UNS S82441 DSS, respectively, verified a similar percentage in weight of the σ phase, that is, 31% Cr, 2% nickel, and 5% molybdenum after treatment at 850 °C, and 32% Cr, 2% Ni, and 9% Mo in the treatment at 930 °C. However, the values obtained are found within a composition band in several studies [22,32,49].

We also observed that even with the presence of the σ phase in the S850, S850-2h, and S950 conditions, the chemical composition of the α and γ phases did not suffer enough alterations to affirm that this deleterious phase influenced the percentage in weight of the elements Cr, Ni, and Mo. The same behavior was observed by Cavalcanti, Muterlle, and Reinke [27] when conducting thermal treatments at 850 °C in the UNS S82441 DSS for 30, 300, and 3000 min, increasing the σ volumetric fraction from around 2% to 20%, without finding alterations in the chemical composition of phases α and γ.

Furthermore, the chemical composition of the ferrite (α) phase in the S850-2h condition could not be measured. This occurred because the ferrite phase, which presented a percentage volume of 7.1 ± 0.8%, found in the boundaries of the σ, was severely damaged, preventing an accurate measurement. Gong et al. [20] also faced similar difficulties when determining the chemical composition of the σ and austenite phases in their study material despite the phases being visible.

Moreover, no significant alteration was observed in the chemical composition after the corrosion tests. The chemical composition of the ferrite, austenite, and σ phases in the investigated conditions remained close to the measures obtained before the DL-EPR tests.

## 4. Conclusions

- The microstructural analysis allowed the following conclusions:

The BM, S1000, and S1150 conditions presented a two-phase structure containing the α (ferrite) and γ (austenite) phases, and the higher the thermal treatment temperature, the higher the phase α percentage volume, and consequently, the lower the percentage volume of the γ phase.

The S850-2h sample showed the highest precipitation kinetics of the σ (20.8 ± 3.4%), followed by the S850 (16.0 ± 3.1%), and the S950 (10.9 ± 2.0%) conditions. The secondary austenite and χ phases were only observed in the S850 condition.

- According to the nanomechanical analysis:

The highest hardness values were obtained for sample S850-2h, which was treated for a longer time and presented a higher fraction of the σ. Hardness mean values (~3.6 GPa for ferrite and austenite, and ~8.9 GPa for σ) and the elasticity modulus (~196, ~198, and ~207 GPa for the austenite, ferrite, and σ phases, respectively) of each phase were compatible with the values found in the literature.

- According to the electrochemical analysis:

The higher the sulfuric acid concentration in the DL-EPR electrolyte, the higher the current observed. In all electrolytes studied, the samples treated at 850 °C (30 min and 2 h) showed the highest corrosion currents.

Although the S950 condition presented the σ, only the samples treated at 850 °C showed intergranular corrosion in the DL-EPR test with H_2_SO_4_ 2.5 mol/L and HCl 1 mol/L electrolyte. This was evidenced by the formation of a third peak in the reactivation curves. The higher resistance of the S950 condition was associated with the lower volumetric fraction of the σ phase, its morphology, and the higher aging temperature.

Two peaks characteristic of dissolution were observed in the DL-EPR activation curves. The lower potential peak was associated with ferrite, while the higher potential was associated with austenite. We also observed, by means of the deconvolutions, a correlation between the current density of each peak and the volumetric fraction of the respective phases.

The EDS results did not reveal significant alterations in ferrite and austenite chemical composition in all tested conditions, including those presenting precipitation of the σ. Likewise, no significant changes were verified after the DL-EPR test in the chemical composition of the phases identified in each sample. 

## Figures and Tables

**Table 1:** Summary of the DL-EPR test parameters reported in the literature to evaluate DSS intragranular corrosion.

DSS	Electrolyte	Surface Preparation Methods	Polarization	Thermal Treatment (Time)	Ref.
SAF 2205	0.5 M H_2_SO_4_ + 0.001 M TA	Paper grit was not informed	−0.5 V until 0.3 V (SCE)	1100 °C (240 min)	[15]
22% Cr	2 M H_2_SO_4_ + 1 M HCl	Grounded until 600-grit paper	−0.5 V until 0.4 V (SCE)	850 °C (2–30 min) and 1070–1150 °C (not informed)	[17]
AISI 2205	2 M H_2_SO_4_ + 1 M HCl	Paper grit was not informed	E*_start_* at OCP * until 0.6 V vs. OCP *	850 °C for 5–60 min	[18]
UNS S31803	2 M H_2_SO_4_ + 0.1, 0.5, 1, and 2 M HCl	Grounded until 1000-grit paper	E*_start_* at OCP * until 0.3 V (SCE)	800 °C for 0.5–48 h	[20]

* OCP: Open Circuit Potential.

**Table 2:** Chemical composition in weight percentage of the UNS S32205 duplex stainless steel used.

Chemical Element	C	Cr	Ni	Mo	Mn	Si	Cu	N	P	S
wt%	0.016	22.458	5.345	3.004	1.691	0.393	0.211	0.146	0.029	0.0004

**Table 3:** Codes identifying the thermal treatment conditions investigated in the UNS S32205 duplex stainless steel.

Identification Code	Study Condition
BM (Base Metal)	Industrial condition (1060 °C for 30 s)
S850	BM + Aging at 850 °C for 30 min
S850-2h	BM + Aging at 850 °C for 2 h
S950	BM + Aging at 950 °C for 30 min
S1000	BM + Solubilization at 1000 °C for 30 min
S1150	BM + Solubilization at 1150 °C for 30 min

**Table 4:** Ferrite and austenite dissolution peaks [mV], versus NHE obtained from the DL-EPR curves of the BM of UNS S32205 DSS and subjected to thermal treatments from 850 to 1150 °C.

Electrolyte		H_2_SO_4_ 0.5 mol/L HCl 1 mol/L		H_2_SO_4_ 1.5 mol/L HCl 1 mol/L		H_2_SO_4_ 2.5 mol/L HCl 1 mol/L	
	Phase	Ferrie	Austenite	Ferrite	Austenite	Ferrite	Austenite
Condition							
BM		−103 ± 8	−45 ± 10	−66 ± 2	−17 ± 2	−50 ± 0	13 ± 2
S850 (Activation)		−79 ± 7	−35 ± 1	−82 ± 5	1 ± 10	26 ± 2	60 ± 18
S850 (Reactivation)		-	-	-	-	−57 ± 8	15 ± 13
S850-2h (Activation)		−95 ± 3	−29 ± 1	−90 ± 8	−10 ± 6	26 ± 6	63 ± 4
S850-2h (Reactivation)		-	-	-	-	−52 ± 3	−2 ± 6
S950		−93 ± 5	−38 ± 6	−67 ± 2	−14 ± 2	−35 ± 9	31 ± 33
S1000		−97 ± 1	−39 ± 1	−76 ± 5	−20 ± 4	−62 ± 10	11 ± 18
S1150		−93 ± 2	−44 ± 8	−61 ± 11	−30 ± 4	−75 ± 7	−10 ± 6

**Table 5:** Degree of sensitization of the UNS S32205 DSS subjected to thermal treatments at 850 °C for 0.5 and 2 h after the DL-EPR test carried out in electrolyte containing H_2_SO_4_ 2.5 mol/L and HCl 1 mol/L.

Sample	DOS (I_r_/I_p_)	Classification
S850	0.174 ± 0.077	Sensitized
S850-2h	0.126 ± 0.096	Sensitized

## Data Availability

The raw data supporting the conclusions of this article will be made available by the authors on request.
